# Experiences with a training DSW knowledge model for early-stage researchers

**DOI:** 10.12688/openreseurope.15609.1

**Published:** 2023-06-19

**Authors:** Marie-Dominique Devignes, Malika Smaïl-Tabbone, Hrishikesh Dhondge, Roswitha Dolcemascolo, Jose Gavaldá-García, R. Anahí Higuera-Rodriguez, Anna Kravchenko, Joel Roca Martínez, Niki Messini, Anna Pérez-Ràfols, Guillermo Pérez Ropero, Luca Sperotto, Isaure Chauvot de Beauchêne, Wim Vranken

**Affiliations:** 1 Université de Lorraine, CNRS, Inria, LORIA, Nancy, F-5400, France; 2 Institute for Integrative Systems Biology (I2SysBio), CSIC - University of Valencia, Paterna, 46980, Spain; 3 Department of Biotechnology, Polytechnic University of Valencia, Valencia, 46022, Spain; 4 Interuniversity Institute of Bioinformatics in Brussels, VUB/ULB, Brussels, 1050, Belgium; 5 Structural Biology Brussels, Vrije Universiteit Brussel, Brussels, 1050, Belgium; 6 Dynamic Biosensors GmbH, Munich, 81379, Germany; 7 Department of Physics, School of Natural Sciences, Technical University of Munich, Garching, 85748, Germany; 8 Department of Bioscience, School of Natural Sciences, Technical University of Munich, Garching, 85748, Germany; 9 Giotto Biotech s.r.l,, Florence, 50019, Italy; 10 Magnetic Resonance Center (CERM), Department of Chemistry “Ugo Schiff”, University of Florence, Florence, 50019, Italy; 11 Department of Chemistry-BMC, Uppsala University, Uppsala, 75123, Sweden; 12 Ridgeview Instruments AB, Uppsala, 75237, Sweden

**Keywords:** Data Management Plan, metadata, student training, FAIR principles, open science, structural bioinformatics, molecular biology.

## Abstract

**Background**: Data management is fast becoming an essential part of scientific practice, driven by open science and FAIR (findable, accessible, interoperable, and reusable) data sharing requirements. Whilst data management plans (DMPs) are clear to data management experts and data stewards, understandings of their purpose and creation are often obscure to the producers of the data, which in academic environments are often PhD students.

**Methods**: Within the RNAct EU Horizon 2020 ITN project, we engaged the 10 RNAct early-stage researchers (ESRs) in a training project aimed at formulating a DMP. To do so, we used the Data Stewardship Wizard (DSW) framework and modified the existing Life Sciences Knowledge Model into a simplified version aimed at training young scientists, with computational or experimental backgrounds, in core data management principles. We collected feedback from the ESRs during this exercise.

**Results**: Here, we introduce our new life-sciences training DMP template for young scientists. We report and discuss our experiences as principal investigators (PIs) and ESRs during this project and address the typical difficulties that are encountered in developing and understanding a DMP.

**Conclusions**: We found that the DS-wizard can also be an appropriate tool for DMP training, to get terminology and concepts across to researchers. A full training in addition requires an upstream step to present basic DMP concepts and a downstream step to publish a dataset in a (public) repository. Overall, the DS-Wizard tool was essential for our DMP training and we hope our efforts can be used in other projects.

## Introduction

During the last decade, Open Science practices have become mainstream, with Open Access publications now common ( Mills, 2020), and data collection and sharing under the FAIR (findable, accessible, interoperable, and reusable) principles ( Jacobsen *et al.*, 2020) strongly encouraged by many research funding agencies ^1^. Nowadays, for most research projects, a data management plan ^2^ (DMP) is required as a formal document that outlines how to handle research data both during research and after the research project is completed. The DMP essentially documents key activities in the research data lifecycle, such as the collection, description, preservation, and access or discovery of data. In brief, it should specify the services and legal support that the project needs to make the data as FAIR and open as possible. Such documentation is crucial for the reproducibility of research results, which is a fundamental precept of scientific investigations. International organisations such as the Research Data Alliance (RDA) host working groups which are trying to define DMP standards, while research funding agencies such as Horizon Europe propose template documents for DMPs. However, researchers that produce data need tools to help them create a DMP, and there are still only a limited number of online tools for filling in a DMP questionnaire, probably due to the lack of standardization. The Opidor DMP tool and the ELIXIR Data Stewardship Wizard are good examples of frameworks that facilitate DMP production.

Nevertheless, the collection of data in a way that enables FAIR data sharing is not trivial, and requires some knowledge of underlying principles of data annotation (metadata) and the overall ways in which data can be organized (formats, storage, etc.). Increasing efforts at academic and research institutions, mainly by employing data stewards to help the producers of the data, as well as upcoming changes in evaluation practices, are enabling FAIR data sharing, but there are still many hurdles present. For example, whilst data stewards can educate scientists in data management principles and help them, the scientist themselves have to understand the data management terminology and aims to a certain extent, so that they can reliably collect and organize relevant data, while remaining motivated to do so. This active participation of scientists is especially relevant as data (storage) is often very domain specific, making it impossible for data stewards to understand all the subtleties and prior practices of each field.

Our goal here is to describe the experience of learning about data management by Early- Stage Researchers (ESRs) in the frame of the RNAct Marie Sklodowska Curie European innovative training network (ITN) ( Gownaris *et al.*, 2022). The RNAct ITN ( https://rnact.eu/) started in 2018 and employed 10 ESRs in research with the main goal being the re-design of RNA Recognition Motif (RRM) protein domains. By investigating how these RRMs bind RNA molecules, through structural biology and bioinformatics approaches, their application in biosensor development and synthetic biology was envisaged. The project employed a mix of synergistic computational and experimental approaches, in a 50/50 ratio, and so encompassed two very different audiences in terms of how data is managed. To address this as part of the project, a data management subcommittee consisting of the principal investigators responsible for RNAct data management (DM PIs; Drs. Devignes, Smaïl-Tabbone, Chauvot de Beauchêne and Vranken) was initiated at the start of the project, with regular meetings held. This subcommittee trained the 10 ESRs on basic and practical data management principles, with the main goal the creation of individual Data Management Plans (DMPs). This process highlighted difficulties in explaining data management: what it means, which resources are available, how related it is to the publication of FAIR data, etc. A key difficulty for our ESRs was, for example, how to describe the data they were producing during their PhD thesis work. Explaining this required an iterative process of providing information about data management, while asking them for relevant information about their own datasets. During this process, we developed a ‘simple’ version of the generic Life Sciences Data Stewardship Wizard (DSW) Knowledge Model ( Pergl *et al.*, 2019). This simplified model is aimed at training young scientists, both with computational and experimental backgrounds, in core data management principles. We collected feedback from the ESRs during this exercise. We here report the experiences of ourselves and the ESRs and introduce our new training life sciences data management plan template for young scientists.

## Methods

### Data Stewardship Wizard (DSW) framework

The DSW framework ( Pergl *et al.*, 2019) has become well known through various working groups of ELIXIR, the European Research Infrastructure for bioinformatics ( Harrow *et al.*, 2021), where it is part of an associated set of tools around FAIR data management ( Wilkinson *et al.*, 2016). It is based on a set of core concepts, with default data management plan questionnaires provided, while still allowing flexibility in customization of these templates for specific purposes. We decided to use DSW based on the three following key features.

1. Possibility to get an «RNAct instance» of the DS-Wizard hosted in the DSW Cloud, thanks to the resources provided by the ELIXIR infrastructure
2. Possibility to modify the default «Life Science DSW Knowledge Model» into an RNAct specific Knowledge Model
3. Possibility for the ESRs to create their own DMP projects based on this model.

**Figure 1.**
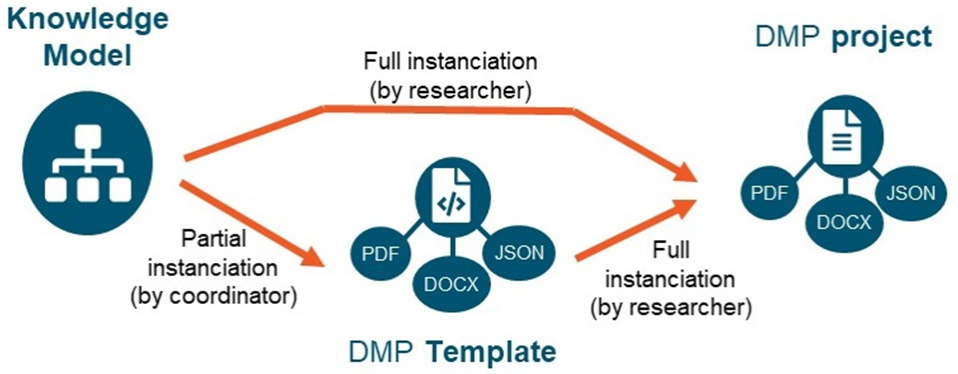
The three levels of DSW framework. The ‘knowledge model’ describes the elements of the DMP in the form of questions, which can be directly answered in by researchers to create a DMP ‘project’, exportable under various formats for various usages. Alternatively, the knowledge model questionnaire can be partially pre-filled by the project coordinator with common information to provide an initial DMP ‘template’ to be further completed by researchers. Permission has been given from the DSW to use their images in this figure.

The DSW framework includes three main levels: knowledge model, template, and project that are schematized in Figure 1.

**Figure 2.**
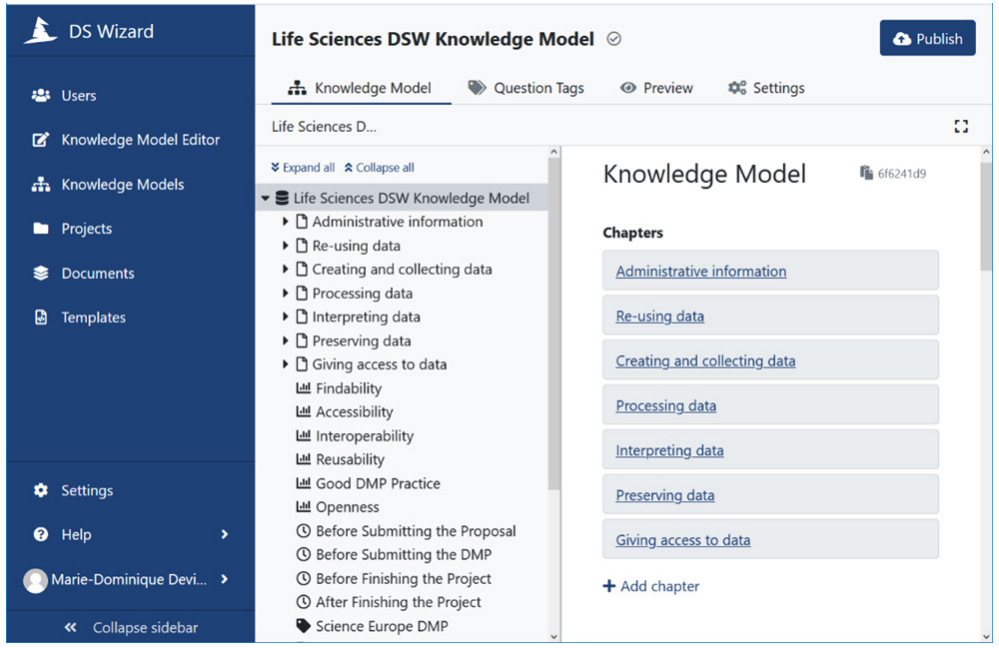
DS Wizard interface for Knowledge Model editor. Permission has been given from the DSW to use their images in this figure.

The DS Wizard tool provides a user-friendly interface for editing the DSW **knowledge model** ( Figure 2). It is organized by chapters, which capture aspects of data management ( *e.g.* Administrative information). Each chapter has sections, which are more specific ( *e.g.* contributors to a DMP) and which contain questions to collect information ( *e.g.* name of a contributor). The questions are tagged depending on the timeline in a data/project lifecycle ( *e.g.* start of the project), and depending on the impact of answers on the compliance with respect to FAIR principles ( *e.g.* answering Yes to the question “Will you describe your data with standard vocabularies or ontologies?” will result in a green FAIR tag).

A knowledge model can be instantiated as a DMP **project** through a user-friendly questionnaire interface. Moreover, one can save a pre-filled version of a project as a **template**, when several DMP projects have to be produced with shared information (e.g. funding sources, licensing modes).

### Other resources

When editing the knowledge model, we added references to the resources and documentation compiled in the ELIXIR RDMkit such as FAIR-SHARING to facilitate the search for relevant ontologies and vocabularies.

### Experimental design

**Figure 3.**
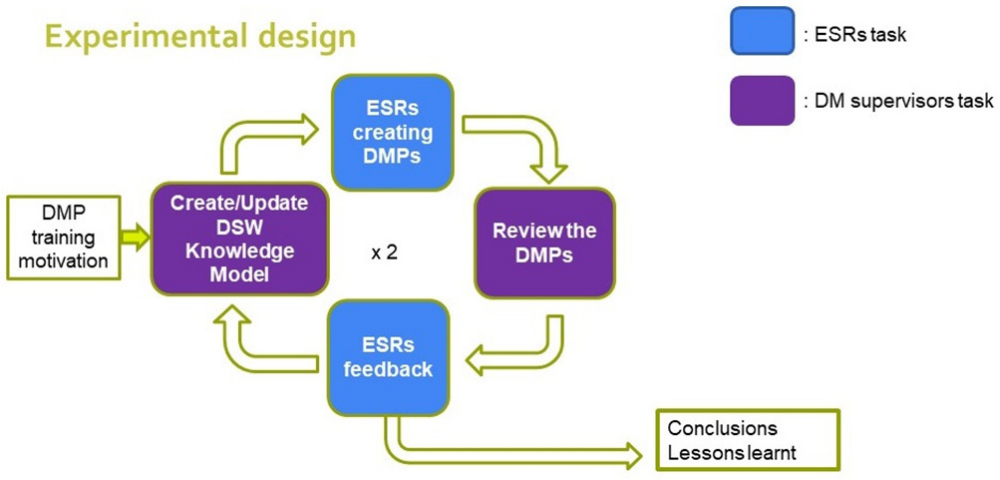
Experimental design for the DMP training. Starting from the modified RNAct knowledge model, ESRs created DMPs that were reviewed, to which ESRs provided further feedback that was taken into account to tune the knowledge model.

The experimental design was based on an iterative process, with task sharing between ESRs and DM PIs as data management supervisors ( Figure 3). All ESRs had to select among all the data they produced one relevant dataset for which they had to produce a DMP project. Starting from the default Life Science DSW knowledge model (version 2.4.0), we performed two iterations of a process composed of four steps: (i) creating or updating the RNAct DSW knowledge model, (ii) having the ESRs create their own DMPs, (iii) reviewing the DMPs and (iv) collecting feedback from the ESRs.

The first round of this process was interactively performed at a hybrid session during an RNAct workshop (3 ^rd^ June 2021, Brussels), with 7 ESRs physically present and 3 present online. For this session, version 1.0.3 of the RNAct_ESRTraining_KM DMP questionnaire was used (see Vranken *et al*., 2023). The following steps were then taken:

1. Presentation of general concepts around data management (available at Vranken *et al*., 2023), including an introduction of the DSW and an overview of the DMP questionnaire.
2. The ESRs selected the dataset(s) that they wanted to create a DMP for, with diverse computational and experimental topics: protein domain structure data; binding and RNA/protein interface data; cell cultures and other data in relation to the Mushashi-1 protein; commercial data; and cell lines (see Table 1).
3. During a 3-hour session they filled in the DMP questionnaire, with technical help provided but minimal help on content, in order to let them independently explore the questionnaire and identify problems.
4. This session was followed by a one-hour session to qualitatively gather their feedback, for example in relation to how much they understood of the DMP terminology that was used, if they encountered specific needs with regard to their dataset, etc.

**Table 1.** Brief description of the datasets addressed by ESRs’ DMPs. Only specific EDAM metadata terms are displayed (3 ^rd^ column). Common terms are RNA and Protein interactions. The full table is available at Vranken *et al*., 2023 (RNA_datasets.xlsx).

ESR	Title	Metadata from EDAM ontology for content description	File formats	Size	Short description
1	Protein Conformational Variability predictions for RRMs	Prediction and recognition; Protein property; Protein folding, stability and design	json	10Mb	Dataset with predictions of the RRM proteins in the InteR3MDB with the ConforMine program.
2	RRM conservation and contact diversity	Sequence alignment analysis (conservation); Protein Structure alignment; Protein-nucleic acid interaction analysis	json, fasta	500Mb	Dataset listing conserved residues and protein- nucleic acid contacts for all positions of the RRM master alignment.
3	Database for RRM-RNA Interactions.	Data integration and warehousing; Residue interaction calculation	sql	500Mb	Complete and comprehensive database about RRMs ( InteR3Mdb)
4	Protein bound ssRNA fragments (PSRNA)	Protein interaction data; Protein binding sites ; Residue interaction calculation	pdb, json	<5 Gb	List of characteristics for protein-RNA contacts extracted from PDB complexes. Each RNA is considered as a set of overlapping trinucleotides.
5	Phage-display results for novel RRM design	Protein-nucleic acid interaction analysis, Protein interaction experiment; Protein interaction data	xlsx	>1Gb	Protein-RNA interaction data produced from processing and analyzing phage display results corresponding to in vitro binding experiments of RNA/ ssDNA to Sex-lethal protein.
6	NMR data analysis for hnRNP A1 protein	Structural biology; NMR; Protein interaction data	xlsx	>1Gb	Data produced from processing and analyzing NMR data collected on hnRNP A1
7	NMR data analysis for Musashi-1 RRM domains	NMR; Protein interaction data	xlsx	>1Gb	Data produced from processing and analyzing NMR data collected on human Musahi-1 protein domains (RRM-1, RRM-2, RRM1-2 and RRM1-2 DM).
8	Fluorometric data on Musashi circuit	Synthetic Biology; Cytometry; Imaging	xlsx	>1Gb	Results of fluorometry on Musashi protein (from Varioskan Lux microplate reader).
9	Human Musashi-1 binding kinetics	Rate of association; Protein- nucleic acid interaction analysis	csv, etbl	>1 Gb	Binding kinetic traces (heliOS) and extracted association and dissociation rate constants, as well as affinity values for a determined protein- nucleic acid interaction (Musashi-1).
10	RNA-Musashi1 interaction in living cells	Protein-nucleic acid interaction analysis; Gene expression	ltr, ltv, txt	<5 Gb	Data collected using LigandTracer technology (.ltr) and analyzed using TraceDrawer (.ltv). Detection of RNA-protein binding and kinetics in living cells using real time binding assays.

We obtained 10 DMPs (available at Vranken *et al*., 2023). This procedure revealed that most ESRs had particular difficulties with: i) the many nested questions inside the DMP questionnaire, pointing to its complexity, and ii) lack of understanding of data management concepts and terminology, especially the metadata section, which was overlooked by most ESRs because they did not comprehend why this was relevant.

**Figure 4.**
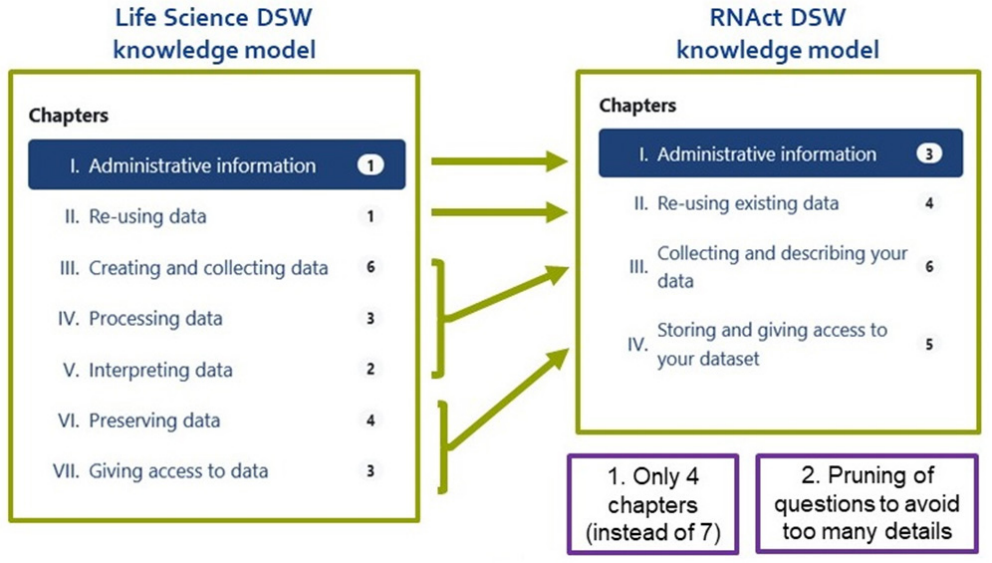
Summary of the initial modifications of the default Life Science DSW model. The number of chapters was reduced and questions were removed and/or simplified.

The qualitative feedback therefore indicated a strong need to simplify the version 1.0.3 knowledge model, with more explanations provided for each of the questions. The 10 DMPs were therefore reviewed in detail by the DM PIs in subsequent meetings and the version 1.0.3 model was updated accordingly to result in the further simplified and annotated RNAct_ESRTraining_KM version 1.0.14 DMP questionnaire (available at Vranken *et al*., 2023) ( Figure 4). A template project based on this questionnaire and pre-filled with general information about the RNAct project (such as funding sources) was proposed to the ESRs in April 2022 (available at Vranken *et al*., 2023). The following steps were then taken:

1. The ESRs were asked to fill this template anew, now online using the – by then more extensive – information about their dataset.
2. To obtain more quantitative feedback, an online survey form (available at Vranken *et al*., 2023) was created for the ESRs to fill in after completing their DMP.

The resulting 8 full completed projects and the survey results were collected in June 2022 (available at Vranken *et al*., 2023). The survey results were qualitatively analysed by the participants in this study.

A preliminary report about this experiment was presented at the DM workshop during the ELIXIR All Hands meeting in June 2022 (Amsterdam, the Netherlands). Final missing ESR information for DMPs was collected separately during December 2022 to obtain 10 complete DMPs, with the data summarized in Table 1.

## Results

### The RNAct DSW Knowledge Model

We present the final RNAct DSW Knowledge Model that was implemented based on the ESR feedback after the first round of DMP production and which is available at https://registry.ds-wizard.org/knowledge-models/rnact:rnact-esr:1.1.0. We reduced the number of chapters from 7 to 4, thereby merging chapters III, IV and V about ‘Creating and Collecting data’, ‘Processing data’, and ‘Interpreting data’ into a single chapter III: ‘Collecting and Describing your data’. We also merged Chapters VI and VII about ‘Preserving data’ and ‘Giving access to data’ into a single chapter IV ‘Storing and giving access to your dataset’.

Meanwhile, we pruned certain nested questions to avoid cognitive overload. We mention here some examples of removed or simplified questions.

- In chapter I, we skipped questions relative to the description of diverse policies and procedures for data management or requiring any additional specialist expertise (as these questions are beyond the ESR role in the RNAct project).
- In chapter II, we merged questions relative to reference and non-reference data;
- In former chapter III, we skipped the questions concerning collaborations with groups/institutions, data integrity, and data integration tools; we also moved the questions relative to file naming/organization to the new chapter IV.
- In former chapter IV, we skipped the questions on how to validate the integrity of the results or plan the computing capacity required for processing data.
- In former chapter V, we removed all questions (e.g. asking for data formats, common ontologies for interpreting the results etc.) as these questions were included in the new chapter III for data description.

**Figure 5.**
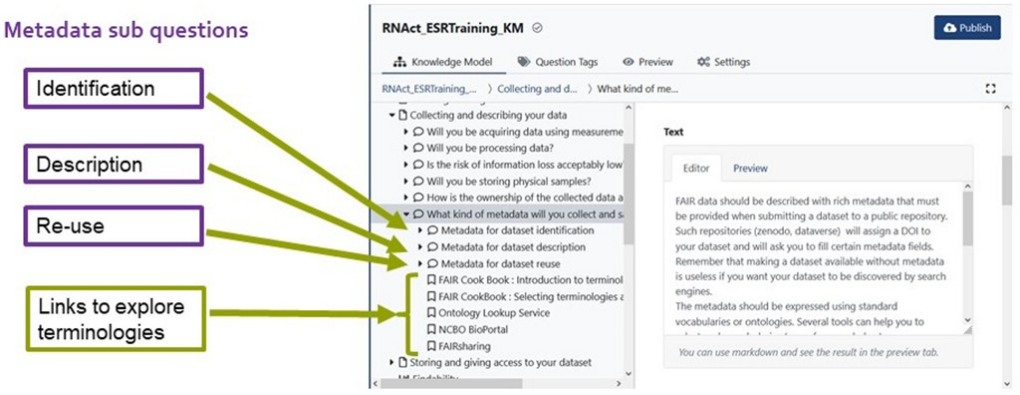
Summary of the structure of the metadata question in the RNAct DSW Knowledge Model. Permission has been given from the DSW to use their images in this figure.

As for the difficulties encountered by ESRs concerning metadata concepts, we tried to explain metadata items by relating them to more familiar concepts, such as items describing published articles in Zotero or BibteX. The original metadata question in the Chapter ‘Collecting and Describing your data’ was restructured as shown in Figure 5. We added three sub-questions to the question “What kind of metadata will you collect and save?” to collect information on metadata for:

- Identifying the dataset
- Describing the dataset
- Reusing the dataset.

By doing so, we wanted to clarify what the metadata concretely refer to. We also provided examples of relevant vocabularies and ontologies for each category of metadata, as well as links to online resources to guide ESRs in the task of understanding and selecting proper vocabulary and ontology terms for their data.

### ESR feedback

The discussions at the DM subcommittee and with the ESRs identified a series of issues that surfaced, and solutions which we employed, which are summarized in Table 2.

**Table 2.** Issues and solutions related to data management identified during the project.

Issue	Possible solutions
The final aim of a DMP is very difficult to get across and is in general considered very abstract, hindering the students’ motivation.	Concrete examples help to clarify why they are creating a DMP, and to motivate them.
The selection of the most important datasets to document and store is difficult, especially during exploratory phases of research. At which point should one archive data?	Provide a wider perspective: which datasets will in the end be most useful for other users? This is especially important for early-stage researchers that might not yet have this view.
It is very difficult to label datasets with metadata and other info in a structured way (using ontologies, …) so that it is easily re-usable. In other words, questions related to terminologies and vocabularies are difficult.	Propose a small concrete list of possible resources, relevant for their research field, from which they may choose.
There are large differences between the level of knowledge about data between ESRs with computational and experimental backgrounds. Both produce and/or work with data, but the way of organizing and labelling data is very different.	Minimum information standards that include both types of information focussed on a research field ( *e.g.* MIADE for intrinsically disordered proteins) are essential to make this practical and easier.
There are large differences between the types of data being used. ‘Large scale’ data are often already in a more consistent digital form, whereas ‘focussed individual projects’ data are often not as well organised, with more freedom available in how to store the data.	In the focussed data case, organised file directory structures are often the key organisational feature, and help to understand dataset content.
The need for correctly licensing data is difficult to get across, but is essential for researchers to understand in relation to making their data open.	Online resources such as http://ufal.github.io/public-license- selector/ to guide the selection of suitable licenses can help.
Once the DMP is finalized, how should the dataset be published in public repositories, and where?	This question goes beyond the DMP production *per se* but it shows that the exercise can prepare to next steps of open science, *e.g.,* data sharing.
The experimentalists need more explanations and (video) tutorials to help them answering the most difficult questions.	Identify existing training material and adapt them to the audience if necessary, *e.g*., from the ELIXIR TESS catalogue

**Figure 6.**
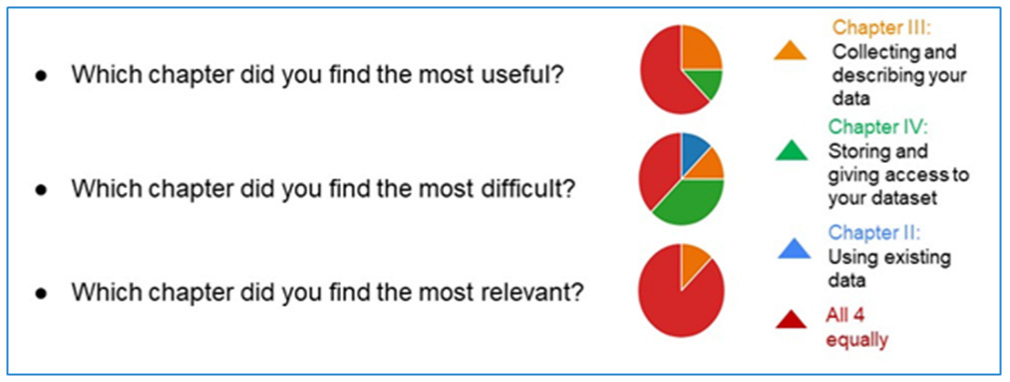
Response distribution for the first three survey questions. The full responses are available from Vranken *et al*., 2023 (RNAct feedback questionnaire.xlsx).

In addition, we formulated a final survey for the ESRs to quantify where the key problems are situated from their perspective. At the end of the process, ESRs were asked to answer this survey. Results for the three checkbox questions are presented in Figure 6.

- To the 1 ^st^ question of the survey, ESRs mostly answered that they found the 4 chapters equally useful, while some of them specified that the most useful was Chapter III (Collecting and describing the data), others opted for Chapter IV (Storing and giving access to the dataset).
- To the 2 ^nd^ question, concerning the most difficult chapters, answers were quite diverse and covered all chapters, except for the first one (administrative information).
- To the 3 ^rd^ question, concerning the relevance of the chapters, most ESRs found that all 4 chapters are equally relevant.

In the survey, we also asked ESRs about what they learnt about data management during the exercise. They answered that they learnt i) good practices for data storage and accessibility, ii) how to use metadata, iii) how important and difficult it is to comply with all data management requirements. To the question about what remained unclear to them, the main issues were related to metadata, with one question about how to publish data in repositories. Finally, the ESRs suggested to further improve the RNAct knowledge model, in particular to include more explanations for experimentalists and video help to answer the most difficult questions.

## Conclusion

A limitation of our study and results is that they only cover in depth the experiences of 10 ESRs in the life sciences field, although with a relatively wide coverage of topics from protein structure to biosensors and synthetic biology, and especially covering both experimental and computational angles. The data we collected is highly qualitative, with some quantitative aspects. The main product of the process is the RNAct knowledge model (version 1.1.0), which we are making available via https://registry.ds-wizard.org/knowledge-models/rnact:rnact-esr:1.1.0, with any updates available via https://registry-ppe.ds-wizard.org/knowledge-models/rnact:rnact-esr:latest. We also noticed that some ESRs filled in the absolute minimum in the templates, likely because of a lack of motivation. Indeed, the DMP exercise remains very abstract as long as there is no way to use the produced DMP as an operational guide for subsequent steps, such as dataset identification, description, storage and publication. This made it difficult to justify the choice of items to keep in the simplified model. The produced DMPs are also not machine actionable; each DMP can be visualized and exported in various formats, but we still lack tools with functionalities such as querying, aggregating, performing statistical analyses, etc. Institutions increasingly ask for DMPs, but their further use beyond the initial generation stage, where it ideally makes researchers ponder their data, seems limited ( Smale *et al.*, 2020). To exploit DMPs as the field progresses, it will become increasingly important to direct researchers to topic specific databases, where their data can be stored in a highly structured and meaningful way. The EOSC ^3^ ( European Open Science Cloud) organisation is working towards this, but combined data storage and DMP infrastructure is required, as ideally the DMP should only describe the specific data locations where field-specific information will be stored. We hope our model will contribute to the mutual understanding between data stewards and researchers that is required to work towards this goal.

In conclusion, we found that the DS-wizard is not only relevant for data stewards, to collect and maintain information on DMPs, but that it is also an appropriate tool for DMP training. This is important, as data management is not an easy task, with the terminology and concepts difficult to get across to researchers. Young people need education on DM at least for at least for their next project submissions and in the more distant future to benefit society as a whole. Both ESRs and DM supervisors learnt a lot during the exercise we describe here, in terms of increased awareness of data management and interoperability requirements. To get a more complete picture of DMP training, we think that such an exercise with DS wizard should be extended with two other steps:

- An upstream step presenting basic concepts and principles on DMPs: such presentations can be selected from existing training material in the ELIXIR TESS catalogue, or can be built by exploring the ELIXIR RDMkit web resources,
- A downstream step that corresponds to the publication of the dataset in a public repository like Zenodo, for which some training material already exists in the FAIR CookBook or in the RDMkit.

Overall, the process we followed thanks to the DS-Wizard played a central role in our DMP training for ESRs in the frame of this RNAct project, and we hope that our efforts can be used in other projects, and by data stewards, to create more complete training on data management for young researchers.

## Consent

All participants gave written informed consent for their participation in the research and for the use and publication of their feedback on the knowledge form. Ethical approval was not required for this research given the non-sensitive nature of the data and the consent and active involvement of the participants in the study.

## Data Availability

Zenodo: Supporting information for the RNAct Data Science Wizard (DSW) knowledge model for early-stage researchers. https://doi.org/10.5281/zenodo.7912419 ( Vranken *et al*., 2023) This project contains the following files: RNAct feedback questionnaire.xlsx RNAct_datasets.xlsx dmp_development.zip Data are available under the terms of the Creative Commons Attribution 4.0 International license (CC-BY 4.0).

## Footnotes

^1^ https://www.e-education.psu.edu/dmpt/node/645

^2^ https://ec.europa.eu/research/participants/docs/h2020-funding-guide/cross-cutting-issues/open-access-data-management/data-management_en.htm

^3^ https://eosc.eu/about-eosc

## References

[ref-1] Gownaris NJ Vermeir K Bittner MI : Barriers to Full Participation in the Open Science Life Cycle among Early Career Researchers. *Data Science Journal.* 2022;21:2. 10.5334/dsj-2022-002

[ref-2] Harrow J Drysdale R Smith A : ELIXIR: Providing a Sustainable Infrastructure for Life Science Data at European Scale. *Bioinformatics.* 2021;37(16):2506–2511. 10.1093/bioinformatics/btab481 34175941 PMC8388016

[ref-3] Jacobsen A de Miranda Azevedo R Juty N : FAIR Principles: Interpretations and Implementation Considerations. *Data Intelligence.* 2020;2(1–2):10–29. 10.1162/dint_r_00024

[ref-4] Mills M : Global trends in open access publication and open data. *J Appl Clin Med Phys.* 2020;21(12):4–5. 10.1002/acm2.13140 33370512 PMC7769382

[ref-5] Pergl R Hooft R Suchánek M : Data Stewardship Wizard: A Tool Bringing Together Researchers, Data Stewards, and Data Experts around Data Management Planning. *Data Science Journal.* 2019;18:59. 10.5334/dsj-2019-059

[ref-6] Smale N Unsworth K Denyer G : A reivew of the History, Advocacy and Efficact of Data Management Plans. *Int J Digit Curation.* 2020;15(1):30. 10.2218/ijdc.v15i1.525

[ref-8] Vranken W Devignes MD Smail M : Supporting information for the RNAct Data Science Wizard (DSW) knowledge model for early-stage researchers.[Dataset]. Zenodo. 2023. 10.5281/zenodo.7912419

[ref-9] Wilkinson MD Dumontier M Aalbersberg IJ : The FAIR Guiding Principles for scientific data management and stewardship. *Sci Data.* 2016;3:160018. 10.1038/sdata.2016.18 26978244 PMC4792175

