# Supplementary material for: Experiences with a training DSW knowledge model for early-stage researchers
Source: Open Res Eur. 2023 Jun 19;3:97. [Version 1] doi: 10.12688/openreseurope.15609.1 (PMC10445825; doi:10.12688/openreseurope.15609.1)
Supplement: Peer review history

# Peer review: Reviewer response for version 1

**DOI:** 10.21956/openreseurope.16870.r33766

**Published:** 2023-08-22

**Authors:** Natalie Meyers

**Affiliations:** 1 University of Notre Dame, Notre Dame, Indiana, USA

The authors describe a unique learning experience wherein Early Stage Researchers (ESRs) from the RNAct EU Horizon 2020 project are offered data stewardship training and then use the Data Stewardship Wizard (DSW) tool for data management planning in a workshop setting. The ESRs encounter the default Life Sciences Knowledge Model (v1.0.3)  when using the Data Stewardship Wizard (DSW) tool which has been used to instantiate a questionnaire that aids participants to prepare data management plans. The ESR participants, each having selected a dataset from diverse RNAct aligned computational and experimental topics, use the questionnaire in the DSW tool to prepare a first round of data management plans for their data sets. Qualitative feedback on the experience is gathered from the ESRs at the end of the workshop to inform a revision of the model (rnact:rnact-esr:1.1.0) in response to ESRs’ usability concerns and better suited to the ESRs' data stewardship skill level.

 About eleven months later the ESRs use the DSW tool again to respond to a new questionnaire (RNAct_ESR-Training_KM v 1.01.4) driven by the revised model. The ESRs use the questionnaire to prepare a second round of data management plans. At the end of the process the ESRs complete a survey to describe what they have learned about data management during the exercise and to provide feedback on using the knowledge model driven questionnaire inside the DSW tool. 

 The project has transparently released as an project output the *RNAct ESR Training KM, *a Data Stewardship Wizard (DSW) knowledge model emphasizing its design as a training tool to help teach early-stage researchers (ESRs) about Data Management Plans (DMP) in the life sciences. The revised model is shared alongside the project’s survey data and its two rounds of DMP output. This sharing is commendable and the outputs should be re-useful to other data stewardship training efforts, to those studying impacts of data management training, as well as for those interested in improving tool usability for authoring data management plans.  

 While the project generated two sets of DMPs (before and after the implementation of rnact:rnact-esr:1.1.0) the write up offers no comparison of the quality of the plans or their likely relative effectiveness in guiding research activity in compliance with funder mandates or relative impact on data management and sharing efficiencies for a project. Such comparisons could be a valuable output given this project's before/after structure. A follow up workshop activity where blinded reviewers rate both sets of DMPs and then unblind to compare the DMP reviews might be an interesting next step.

 In the conclusion, the authors state the “DMP exercise remains very abstract so long as there is no way to use the produced DMP as an operational guide for subsequent steps, such as data set identification, description, storage and publication.” This finding is untestable through the project’s method, and the claim itself not clearly justified by the shared project outputs therefore it should be further explicated for the reader.

 The claim perhaps does not take enough into account how research projects can benefit when their DMPs are pre-populated with explicit targets defined by URLs and/or DOIs  to indicate where a proposed project will do its future data storage & sharing, or how a lab can save time and error when a project's DMP explicitly states how outputs will be licensed. Such pre-declarations or DMP details can in turn be implemented as project defaults that streamline upload activities and assignment of preferred licenses at later time of sharing.

 In the conclusion section it is also stated that  “ . . . the DMP should only describe the specific data locations where field-specific information will be stored.”  This claim is offered in the context of how it is likely to "become increasingly important to direct researchers to topic specific databases, where their data can be stored in a highly structured and meaningful way.” 

 This position in and of itself is untestable through the project’s method and can’t be justified through the shared results. Some further clarity is needed here, because if strictly interpreted or taken out of context this position could significantly narrow the policy purposes of data management planning.  

 Many data management and sharing policies require DMP elements that describe: 1) related tools, software and code; 2) timelines for when and how long data will be available; 3) access, distribution, and re-use considerations, as well as; 4) details surrounding how plan compliance will be monitored and by whom; therefore a DMP compliant with such mandates must include more content than “specific data l;cations where field specific information will be stored”.

 The paper can be improved by grammatical attention to the repeated “at least for at least” phrase in the second paragraph of the conclusion which reads: “Young people need education on DM at least for at least for their next project submissions and in the more distant future to benefit society as a whole.”

 With attention to these few areas of concern this paper is approvable.

Is the study design appropriate and does the work have academic merit?

Yes

Is the work clearly and accurately presented and does it cite the current literature?

Yes

If applicable, is the statistical analysis and its interpretation appropriate?

Not applicable

Are all the source data underlying the results available to ensure full reproducibility?

Yes

Are the conclusions drawn adequately supported by the results?

Partly

Are sufficient details of methods and analysis provided to allow replication by others?

Yes

Reviewer Expertise:

Data stewardship for reproducible model driven research.

I confirm that I have read this submission and believe that I have an appropriate level of expertise to confirm that it is of an acceptable scientific standard, however I have significant reservations, as outlined above.

# Peer review: Reviewer response for version 1

**DOI:** 10.21956/openreseurope.16870.r33767

**Published:** 2023-08-11

**Authors:** Christine R. Kirkpatrick

**Affiliations:** 1 UC San Diego Foundation, La Jolla, California, USA

This article describes a process of engaging with early career researchers through the Data Stewardship Framework to create a data management plan. The use of a knowledge model framework where questions are decided upon and then used by the cohort is an excellent approach, and they build upon prior scholarship and questions. The discussion of how they simplified the model as well as provide more explanation as needed for the target audience is useful and an exercise others will follow.

 I read this article with great interest and found it contributed knowledge that those in the applicable fields will find useful. The conclusions are particularly helpful and timely, including the identification of perquisite knowledge needed to be successful, as well as training on how to prepare data for depositing in a community repository. I look forward to its indexing, so I can cite it and discuss with colleagues.

 Specific suggestions:

- In the abstract, “Data management is fast becoming an essential part of scientific practice,” one could argue it’s been there the whole time since early astronomical observations, Mendel, etc. It hasn’t always been done well or been called out. I’d argue the practice of data management co-evolved with digital data’s emergence. Perhaps change the phrasing here so one isn’t disagreeing with tiny things before they read the major ideas.
- Consider revising, “During the last decade, Open Science practices have become mainstream,”  Perhaps the discussion or prioritization has reached the mainstream, but how far that translates into practice is up for debate. I’d sidestep this normative claim and rephrase
- I found the leap of creating a DMP for a dataset confusing vs. an entire project. If it’s for an existing dataset, is the DMP a hypothetical exercise as if you were creating the dataset? Perhaps 1-2 more sentences in the introduction explaining for the non-Biologist how this represents a typical workflow would be helpful. I apologize if this is explained, and I just didn’t get it. It might be a difference of terms and adding more explanation will make it clearer for non-European audiences.
- The issue claimed in Table 2 seems like a leap. Perhaps this is the case for the data examined? “There are large differences between the types of data being used. ‘Large scale’ data are often already in a more consistent digital form, whereas ‘focussed individual projects’ data are often not as well organised, with more freedom available in how to store the data.
-  I would have liked to see more than an assumption here, but evidence for this guess: “We also noticed that some ESRs filled in the absolute minimum in the templates, *likely because of a lack of motivation*.”

I believe all the data is available as described in the paper, but I could only find the knowledge model files and PDFs. I wasn't sure where the survey raw data was.

Is the study design appropriate and does the work have academic merit?

Yes

Is the work clearly and accurately presented and does it cite the current literature?

Yes

If applicable, is the statistical analysis and its interpretation appropriate?

Yes

Are all the source data underlying the results available to ensure full reproducibility?

Partly

Are the conclusions drawn adequately supported by the results?

Partly

Are sufficient details of methods and analysis provided to allow replication by others?

Yes

Reviewer Expertise:

Data management, computer science

I confirm that I have read this submission and believe that I have an appropriate level of expertise to confirm that it is of an acceptable scientific standard.

# Peer review: Reviewer response for version 1

**DOI:** 10.21956/openreseurope.16870.r33768

**Published:** 2023-07-26

**Authors:** Rob Hooft

**Affiliations:** 1 Architecture and Implementation, Health-RI, Utrecht, The Netherlands; 2 Dutch Techcentre for Life Sciences, Utrecht, The Netherlands

The work is an interesting combination of a short feedback improvement cycle for a Data Stewardship questionnaire and a training for early career researchers on data stewardship. This concept of collecting feedback from people who are new to the subject of data stewardship is very interesting, and using it immediately to improve the structure and content of the questionnaire is valuable. It is an interesting puzzle to balance the goals of complete understanding of the questions by the trainees and improvement of the knowledge model.

 I do have some questions about the execution of the changes in the knowledge model that could be valuable to address in the paper. The authors have taken different approaches on different parts of the feedback: for some of the concepts that the trainees found complicated or confusing the solution has been to take them out of the knowledge model, where for other concepts, like metadata, they have restructured and augmented the knowledge model.

 In general, the DSW was created with the idea that by incorporating expertise from different areas of data science and different branches of science into a single model, we could all learn to do things better.

- Some concepts may be applicable only to part of the audience who is doing data stewardship planning. However, I think that a better approach than to remove such subjects from a derived knowledge model would be to structure the knowledge model in such a way that non-applicable concepts are “hidden” between questions that are identifying their relevance.
- Other concepts may be difficult to grasp. Here, a similar point applies: difficult concepts should not be avoided, but possibly better explained and supported by links to training materials.

It is clear that not all branches can get equal attention in a process as described in this paper, but is removal of topics the best approach?

 Regarding the restructuring of the chapters I would generally like to see some solid input from teachers of data stewardship on what version of the life cycle works best to bring across the topic. The existing chapters in the core knowledge model were aligned as much as possible to the stages in the data life cycle from the UK Data Service, which is a data life cycle (among many) that is frequently re-used and built upon. I would really hesitate to "chop" chapters out in general.

 The development of tailored, pruned, knowledge models for specific audiences are often a first reaction to the broad data stewardship approach in DS Wizard. The experience gained by the authors of this paper is very valuable. However, it is my opinion that the highest value is obtained if the single “core” knowledge model of the DS Wizard captures as much as possible of diverse expertise. For example, I really like the way the “metadata” branch of the knowledge model was developed in the work described in this paper. The incorporation of the new extended metadata section into the core model would give it a larger exposure, and thereby benefit to a larger group of future research project. I am looking forward to working with the authors of this paper to see how we can achieve that goal together.

 Some textual remarks:

- Introduction, paragraph 2: “requires some knowledge of underlying principles of data annotation …. organized”. I’m looking for a connection between this knowledge and the tool used for data stewardship planning.  In your opinion, is it enough knowledge if the researcher is consciously incompetent about data stewardship? And could that state of “conscious incompetence” be achieved using the DS Wizard?
- Figure 1: “instanciation” should be “instantiation”.
- Under Figure 1: “questions are tagged depending on the timeline in a data/project lifecycle” -> not timeline, but time point in the project lifecycle by when they should probably be answered.
- “answering Yes to the question “Will you describe your data with standard vocabularies or ontologies?” will result in a green FAIR tag” -> Can this be made a little bit more precise? The flag is already visible before the answer is given, because it is not an exam on FAIR knowledge, the flags are part of the guidance. The particular green flag in this example is “interoperability”.
- Under figure 2 “save a pre-filled version”; I would state “partially pre-filled”.
- The paper systematically talks about “DMP Projects”, we as authors of DS Wizard usually think in the direction of a “Project DMP” where the DMP is not a separate project, but serving a research project.
- Table 2: I usually try to inspire motivation to make a DMP through stories like irritant things that everyone encounters when dealing with data and that could be avoided using proper planning. An alternative is a more comprehensive story like the famous “panda movie” (search youtube for “data sharing and management snafu in 3 short acts”).
- Table 2: Expresses the need for video instruction. I’d like to express my agreement there.

In conclusion: I am very happy to read that DSW is suitable for training purposes. In the described work this is done in a “supervised” way with DSW in a co-functioning role with human experts; but the work described works on making the DSW tool more usable for self-education too.

Is the study design appropriate and does the work have academic merit?

Yes

Is the work clearly and accurately presented and does it cite the current literature?

Yes

If applicable, is the statistical analysis and its interpretation appropriate?

Not applicable

Are all the source data underlying the results available to ensure full reproducibility?

Yes

Are the conclusions drawn adequately supported by the results?

Partly

Are sufficient details of methods and analysis provided to allow replication by others?

Yes

Reviewer Expertise:

I am one of the authors of the Data Stewardship Wizard, and engineer of the first versions of the Core Knowledge model. I am not an expert in training.

I confirm that I have read this submission and believe that I have an appropriate level of expertise to confirm that it is of an acceptable scientific standard, however I have significant reservations, as outlined above.

# Peer review: Reviewer response for version 1

**DOI:** 10.21956/openreseurope.16870.r33051

**Published:** 2023-07-18

**Authors:** Fotis Psomopoulos

**Affiliations:** 1 Institute of Applied Biosciences, Centre of Research and Technology Hellas, Thessaloniki, Greece

The work presented here is an outcome of the RNAct project towards assessing data management challenges for early-stage researchers (ESRs). Ten ESRs from life sciences were trained on data management principles with the goal of creating individual Data Management Plans (DMPs). However, even the process of explaining data management and its relation to FAIR data publication had it's own challenges, especially when ESRs were asked to describe their own datasets. To overcome this, a simplified version of the Life Sciences Data Stewardship Wizard (DSW) Knowledge Model was developed, as a means towards offering training in data management. ESRs were asked to provide feedback on the use of the DSW, and based on their feedback, a new training template for life sciences data management plans was designed. The experimental approach towards the design of this new and simplified DMP was iterative, with revisions between feedback from the ESRs and re-design from the data management supervisors.

 Overall the work presented here is interesting, and highlights (among others) the need to offer targeted training on data management and DMPs, especially for early career researchers. Some challenges are pervasive (such as insufficient motivation to feel in the DMP, or the lack of specificity of the DMP questions), but the work here established the impact a simplified version of a DMP might have.

 The work is clearly and accurately presented, and there are sufficient citations on the current literature. The study design, although minimal and fairly limited in size and scope (10 ESRs from a subdomain within Life Sciences), is appropriate given that the challenges of DMPs are rather interdisciplinary.

 Due to the nature of the work, it's rather hard to replicate the analysis - it would be recommended to provide some suggestions (or recipes) on how the overall approach could be reproduced in another domain or, ideally, generalized for a wider audience. Moreover, it would be also useful to provide some brief rationale on the design choices for the simplified model (e.g. given that a challenge is lack of specificity in the DMP questions, why were the tools for data integrity skipped and not replaced by a life-science specific list of "relevant" tools, etc).

 The sharing of the data and the models is exemplary, deposited to zenodo with sufficient metadata to be reused in future studies.

Is the study design appropriate and does the work have academic merit?

Yes

Is the work clearly and accurately presented and does it cite the current literature?

Yes

If applicable, is the statistical analysis and its interpretation appropriate?

Not applicable

Are all the source data underlying the results available to ensure full reproducibility?

Yes

Are the conclusions drawn adequately supported by the results?

Partly

Are sufficient details of methods and analysis provided to allow replication by others?

Partly

Reviewer Expertise:

Training, Software Management

I confirm that I have read this submission and believe that I have an appropriate level of expertise to confirm that it is of an acceptable scientific standard, however I have significant reservations, as outlined above.
